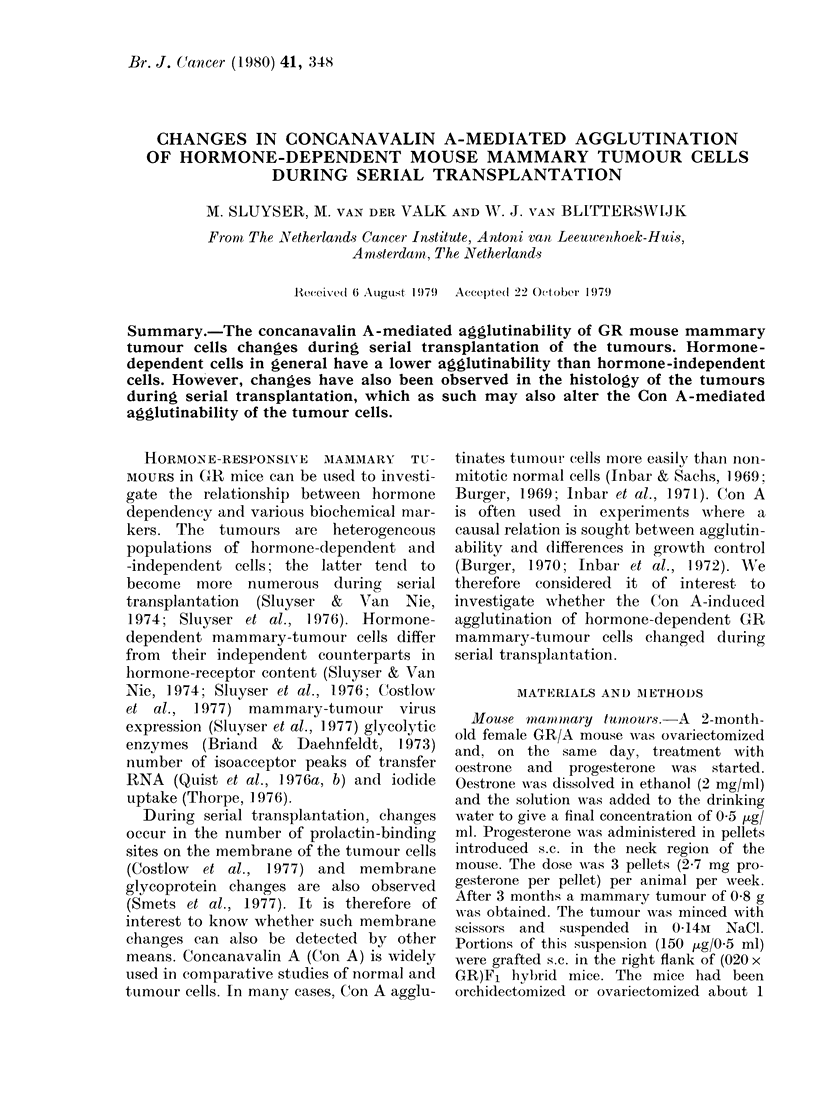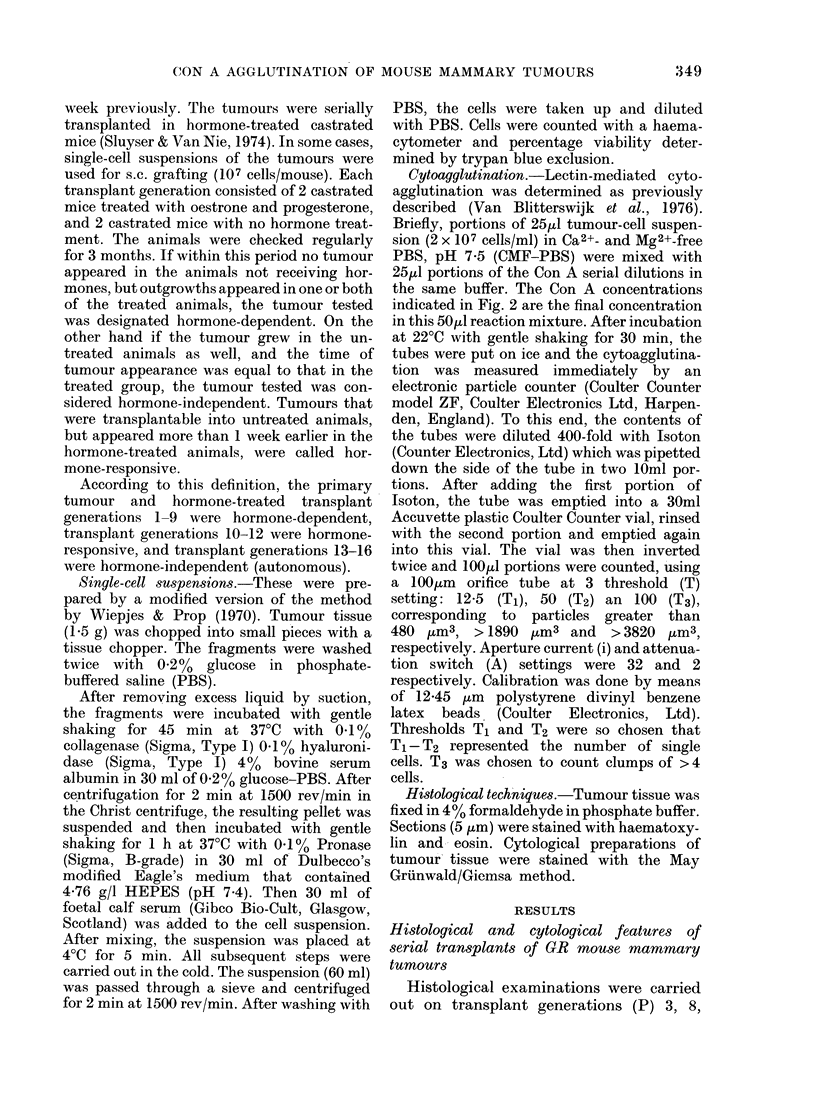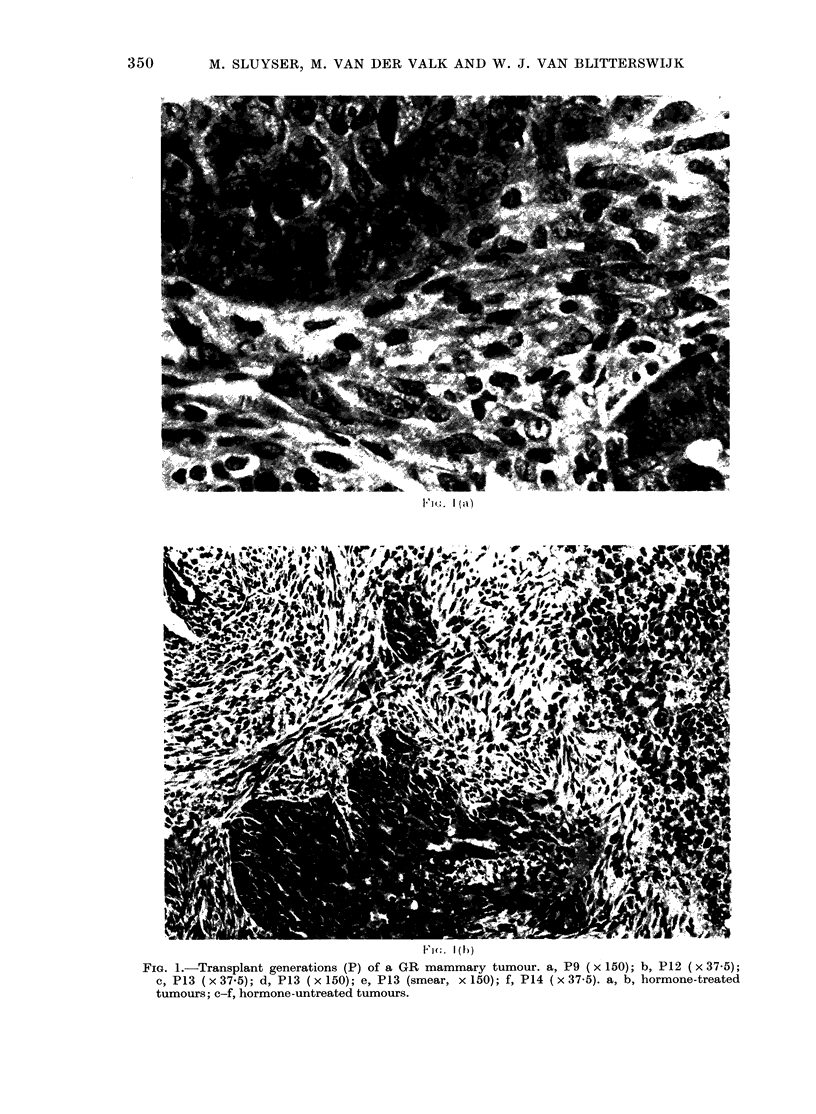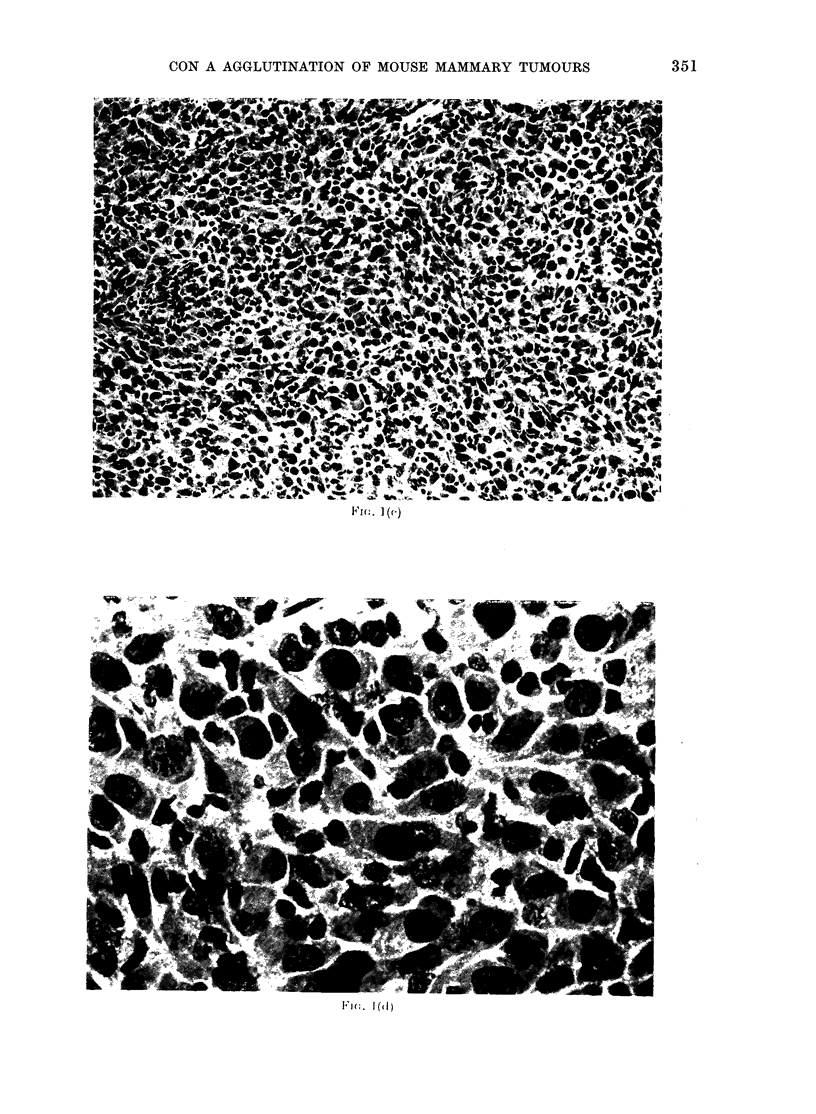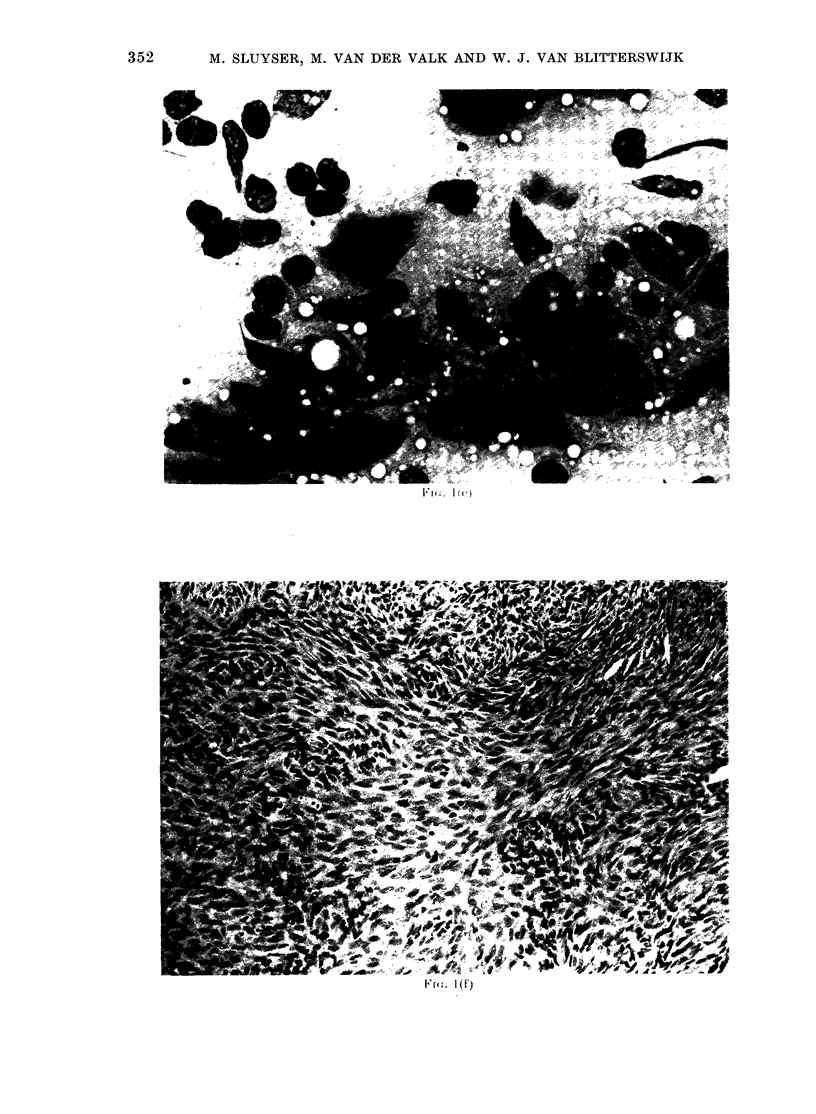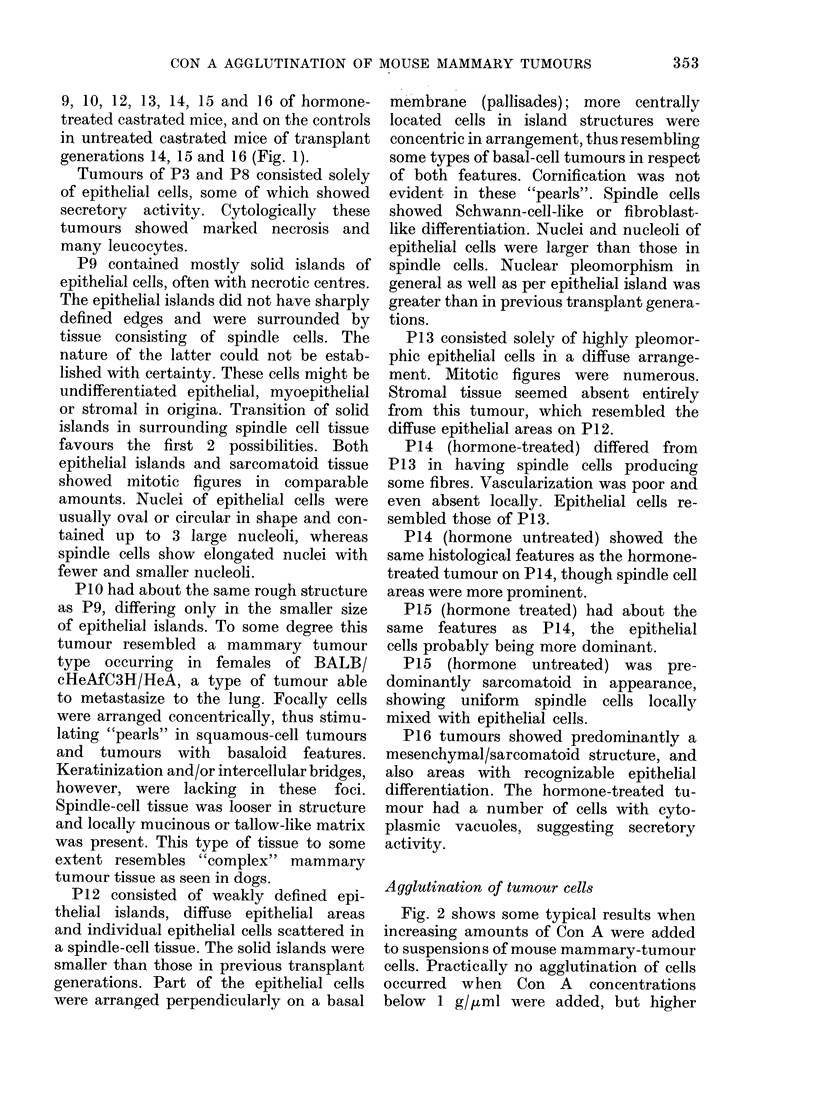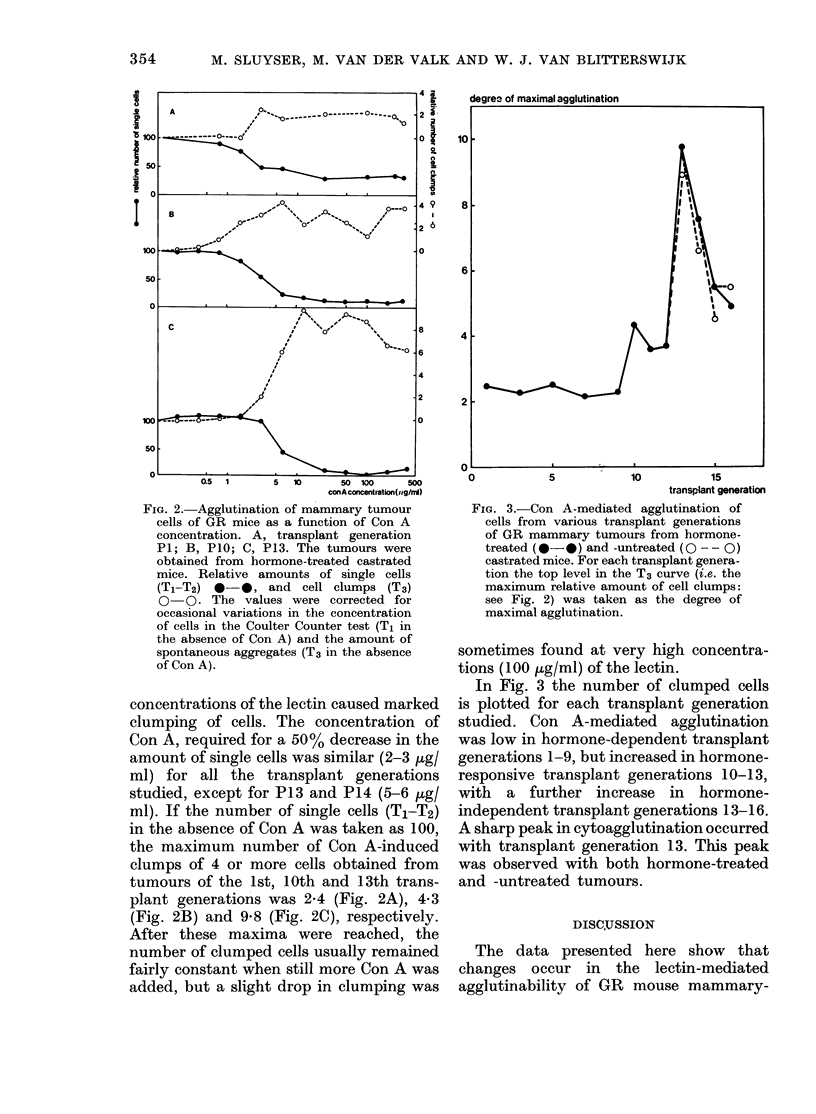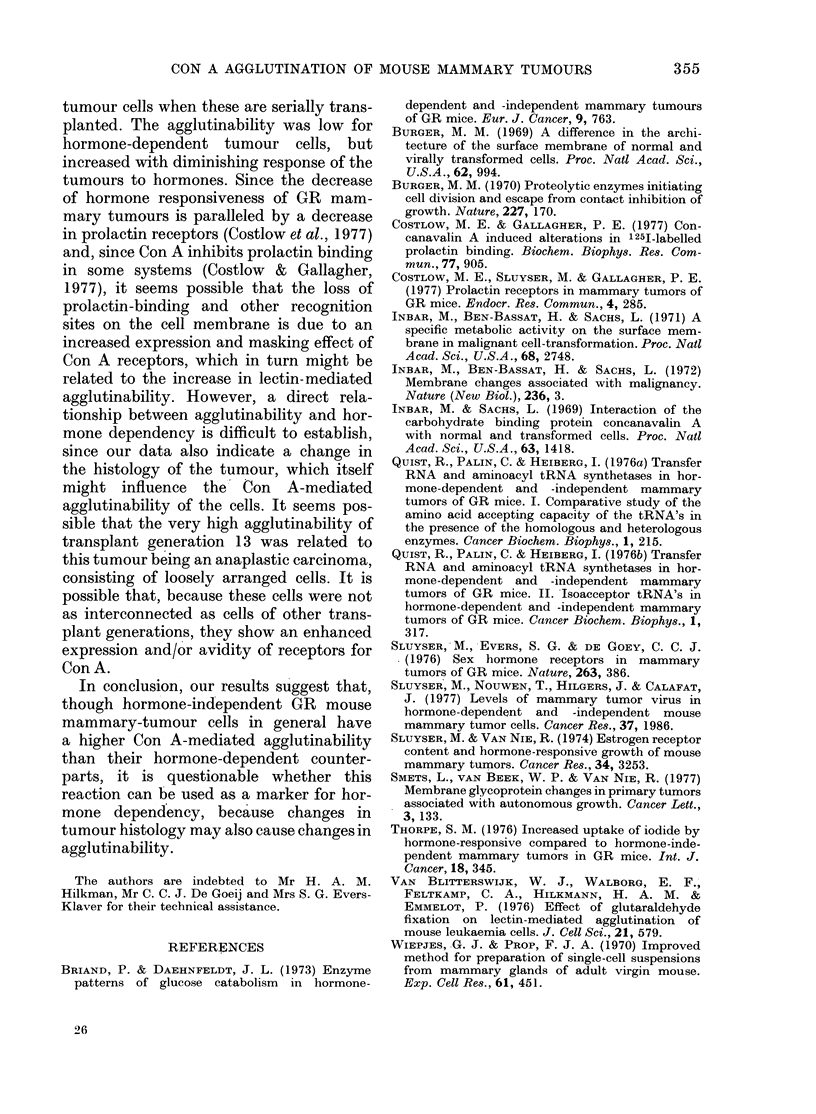# Changes in concanavalin A-mediated agglutination of hormone-dependent mouse mammary tumour cells during serial transplantation.

**DOI:** 10.1038/bjc.1980.57

**Published:** 1980-03

**Authors:** M. Sluyser, M. van der Valk, W. J. van Blitterswijk

## Abstract

**Images:**


Br. J. Cancer (1980) 41, 348

### CHANGES IN CONCANAVALIN A-MEDIATED AGGLUTINATION OF HORMONE-DEPENDENT MOUSE MAMMARY TUMOUR CELLS DURING SERIAL TRANSPLANTATION

M. SLUYSER, M. VAN DER VALK AND W. J. VAN BLITTERSWIJK

*From The Netherlands Cancer Institute, Antoni van Leeuwenhoek-Huis, Amsterdam, The Netherlands*

Received 6 August 1979 Accepted 22 October 1979

**Summary.—The concanavalin A-mediated agglutinability of GR mouse mammary tumour cells changes during serial transplantation of the tumours. Hormone-dependent cells in general have a lower agglutinability than hormone-independent cells. However, changes have also been observed in the histology of the tumours during serial transplantation, which as such may also alter the Con A-mediated agglutinability of the tumour cells.**

HORMONE-RESPONSIVE MAMMARY TUMOURS in GR mice can be used to investigate the relationship between hormone dependency and various biochemical markers. The tumours are heterogeneous populations of hormone-dependent and -independent cells; the latter tend to become more numerous during serial transplantation (Sluyser & Van Nie, 1974; Sluyser *et al.*, 1976). Hormone-dependent mammary-tumour cells differ from their independent counterparts in hormone-receptor content (Sluyser & Van Nie, 1974; Sluyser *et al.*, 1976; Costlow *et al.*, 1977) mammary-tumour virus expression (Sluyser *et al.*, 1977) glycolytic enzymes (Briand & Daehnfeldt, 1973) number of isoacceptor peaks of transfer RNA (Quist *et al.*, 1976*a*, *b*) and iodide uptake (Thorpe, 1976).

During serial transplantation, changes occur in the number of prolactin-binding sites on the membrane of the tumour cells (Costlow *et al.*, 1977) and membrane glycoprotein changes are also observed (Smets *et al.*, 1977). It is therefore of interest to know whether such membrane changes can also be detected by other means. Concanavalin A (Con A) is widely used in comparative studies of normal and tumour cells. In many cases, Con A agglutinates tumour cells more easily than non-mitotic normal cells (Inbar & Sachs, 1969; Burger, 1969; Inbar *et al.*, 1971). Con A is often used in experiments where a causal relation is sought between agglutinability and differences in growth control (Burger, 1970; Inbar *et al.*, 1972). We therefore considered it of interest to investigate whether the Con A-induced agglutination of hormone-dependent GR mammary-tumour cells changed during serial transplantation.

### MATERIALS AND METHODS

*Mouse mammary tumours.*—A 2-month-old female GR/A mouse was ovariectomized and, on the same day, treatment with oestrone and progesterone was started. Oestrone was dissolved in ethanol (2 mg/ml) and the solution was added to the drinking water to give a final concentration of 0·5 μg/ml. Progesterone was administered in pellets introduced s.c. in the neck region of the mouse. The dose was 3 pellets (2·7 mg progesterone per pellet) per animal per week. After 3 months a mammary tumour of 0·8 g was obtained. The tumour was minced with scissors and suspended in 0·14M NaCl. Portions of this suspension (150 μg/0·5 ml) were grafted s.c. in the right flank of (020 × GR)$F_1$ hybrid mice. The mice had been orchidectomized or ovariectomized about 1

week previously. The tumours were serially transplanted in hormone-treated castrated mice (Sluyser & Van Nie, 1974). In some cases, single-cell suspensions of the tumours were used for s.c. grafting ($10^7$ cells/mouse). Each transplant generation consisted of 2 castrated mice treated with oestrone and progesterone, and 2 castrated mice with no hormone treatment. The animals were checked regularly for 3 months. If within this period no tumour appeared in the animals not receiving hormones, but outgrowths appeared in one or both of the treated animals, the tumour tested was designated hormone-dependent. On the other hand if the tumour grew in the untreated animals as well, and the time of tumour appearance was equal to that in the treated group, the tumour tested was considered hormone-independent. Tumours that were transplantable into untreated animals, but appeared more than 1 week earlier in the hormone-treated animals, were called hormone-responsive.

According to this definition, the primary tumour and hormone-treated transplant generations 1–9 were hormone-dependent, transplant generations 10–12 were hormone-responsive, and transplant generations 13–16 were hormone-independent (autonomous).

*Single-cell suspensions.*—These were prepared by a modified version of the method by Wiepjes & Prop (1970). Tumour tissue (1·5 g) was chopped into small pieces with a tissue chopper. The fragments were washed twice with 0·2% glucose in phosphate-buffered saline (PBS).

After removing excess liquid by suction, the fragments were incubated with gentle shaking for 45 min at 37°C with 0·1% collagenase (Sigma, Type I) 0·1% hyaluronidase (Sigma, Type I) 4% bovine serum albumin in 30 ml of 0·2% glucose–PBS. After centrifugation for 2 min at 1500 rev/min in the Christ centrifuge, the resulting pellet was suspended and then incubated with gentle shaking for 1 h at 37°C with 0·1% Pronase (Sigma, B-grade) in 30 ml of Dulbecco's modified Eagle's medium that contained 4·76 g/l HEPES (pH 7·4). Then 30 ml of foetal calf serum (Gibco Bio-Cult, Glasgow, Scotland) was added to the cell suspension. After mixing, the suspension was placed at 4°C for 5 min. All subsequent steps were carried out in the cold. The suspension (60 ml) was passed through a sieve and centrifuged for 2 min at 1500 rev/min. After washing with PBS, the cells were taken up and diluted with PBS. Cells were counted with a haemacytometer and percentage viability determined by trypan blue exclusion.

*Cytoagglutination.*—Lectin-mediated cytoagglutination was determined as previously described (Van Blitterswijk *et al.*, 1976). Briefly, portions of 25$\mu$l tumour-cell suspension ($2 \times 10^7$ cells/ml) in $Ca^{2+}$- and $Mg^{2+}$-free PBS, pH 7·5 (CMF–PBS) were mixed with 25$\mu$l portions of the Con A serial dilutions in the same buffer. The Con A concentrations indicated in Fig. 2 are the final concentration in this 50$\mu$l reaction mixture. After incubation at 22°C with gentle shaking for 30 min, the tubes were put on ice and the cytoagglutination was measured immediately by an electronic particle counter (Coulter Counter model ZF, Coulter Electronics Ltd, Harpenden, England). To this end, the contents of the tubes were diluted 400-fold with Isoton (Counter Electronics, Ltd) which was pipetted down the side of the tube in two 10ml portions. After adding the first portion of Isoton, the tube was emptied into a 30ml Accuvette plastic Coulter Counter vial, rinsed with the second portion and emptied again into this vial. The vial was then inverted twice and 100$\mu$l portions were counted, using a 100$\mu$m orifice tube at 3 threshold (T) setting: 12·5 ($T_1$), 50 ($T_2$) an 100 ($T_3$), corresponding to particles greater than 480 $\mu m^3$, $>1890$ $\mu m^3$ and $>3820$ $\mu m^3$, respectively. Aperture current (i) and attenuation switch (A) settings were 32 and 2 respectively. Calibration was done by means of 12·45 $\mu$m polystyrene divinyl benzene latex beads (Coulter Electronics, Ltd). Thresholds $T_1$ and $T_2$ were so chosen that $T_1 - T_2$ represented the number of single cells. $T_3$ was chosen to count clumps of $>4$ cells.

*Histological techniques.*—Tumour tissue was fixed in 4% formaldehyde in phosphate buffer. Sections (5 $\mu$m) were stained with haematoxylin and eosin. Cytological preparations of tumour tissue were stained with the May Grünwald/Giemsa method.

### RESULTS

### *Histological and cytological features of serial transplants of GR mouse mammary tumours*

Histological examinations were carried out on transplant generations (P) 3, 8,

FIG. 1(a)

FIG. 1(b)

FIG. 1.—Transplant generations (P) of a GR mammary tumour. a, P9 (×150); b, P12 (×37·5); c, P13 (×37·5); d, P13 (×150); e, P13 (smear, ×150); f, P14 (×37·5). a, b, hormone-treated tumours; c–f, hormone-untreated tumours.

FIG. 1(c)

FIG. 1(d)

FIG. 1(e)

FIG. 1(f)

9, 10, 12, 13, 14, 15 and 16 of hormone-treated castrated mice, and on the controls in untreated castrated mice of transplant generations 14, 15 and 16 (Fig. 1).

Tumours of P3 and P8 consisted solely of epithelial cells, some of which showed secretory activity. Cytologically these tumours showed marked necrosis and many leucocytes.

P9 contained mostly solid islands of epithelial cells, often with necrotic centres. The epithelial islands did not have sharply defined edges and were surrounded by tissue consisting of spindle cells. The nature of the latter could not be established with certainty. These cells might be undifferentiated epithelial, myoepithelial or stromal in origina. Transition of solid islands in surrounding spindle cell tissue favours the first 2 possibilities. Both epithelial islands and sarcomatoid tissue showed mitotic figures in comparable amounts. Nuclei of epithelial cells were usually oval or circular in shape and contained up to 3 large nucleoli, whereas spindle cells show elongated nuclei with fewer and smaller nucleoli.

P10 had about the same rough structure as P9, differing only in the smaller size of epithelial islands. To some degree this tumour resembled a mammary tumour type occurring in females of BALB/cHeAfC3H/HeA, a type of tumour able to metastasize to the lung. Focally cells were arranged concentrically, thus stimulating "pearls" in squamous-cell tumours and tumours with basaloid features. Keratinization and/or intercellular bridges, however, were lacking in these foci. Spindle-cell tissue was looser in structure and locally mucinous or tallow-like matrix was present. This type of tissue to some extent resembles "complex" mammary tumour tissue as seen in dogs.

P12 consisted of weakly defined epithelial islands, diffuse epithelial areas and individual epithelial cells scattered in a spindle-cell tissue. The solid islands were smaller than those in previous transplant generations. Part of the epithelial cells were arranged perpendicularly on a basal membrane (pallisades); more centrally located cells in island structures were concentric in arrangement, thus resembling some types of basal-cell tumours in respect of both features. Cornification was not evident in these "pearls". Spindle cells showed Schwann-cell-like or fibroblast-like differentiation. Nuclei and nucleoli of epithelial cells were larger than those in spindle cells. Nuclear pleomorphism in general as well as per epithelial island was greater than in previous transplant generations.

P13 consisted solely of highly pleomorphic epithelial cells in a diffuse arrangement. Mitotic figures were numerous. Stromal tissue seemed absent entirely from this tumour, which resembled the diffuse epithelial areas on P12.

P14 (hormone-treated) differed from P13 in having spindle cells producing some fibres. Vascularization was poor and even absent locally. Epithelial cells resembled those of P13.

P14 (hormone untreated) showed the same histological features as the hormone-treated tumour on P14, though spindle cell areas were more prominent.

P15 (hormone treated) had about the same features as P14, the epithelial cells probably being more dominant.

P15 (hormone untreated) was predominantly sarcomatoid in appearance, showing uniform spindle cells locally mixed with epithelial cells.

P16 tumours showed predominantly a mesenchymal/sarcomatoid structure, and also areas with recognizable epithelial differentiation. The hormone-treated tumour had a number of cells with cytoplasmic vacuoles, suggesting secretory activity.

*Agglutination of tumour cells*

Fig. 2 shows some typical results when increasing amounts of Con A were added to suspensions of mouse mammary-tumour cells. Practically no agglutination of cells occurred when Con A concentrations below 1 g/$\mu$ml were added, but higher

Fig. 2.—Agglutination of mammary tumour cells of GR mice as a function of Con A concentration. A, transplant generation P1; B, P10; C, P13. The tumours were obtained from hormone-treated castrated mice. Relative amounts of single cells ($T_1$–$T_2$) ●—●, and cell clumps ($T_3$) ○—○. The values were corrected for occasional variations in the concentration of cells in the Coulter Counter test ($T_1$ in the absence of Con A) and the amount of spontaneous aggregates ($T_3$ in the absence of Con A).

Fig. 3.—Con A-mediated agglutination of cells from various transplant generations of GR mammary tumours from hormone-treated (●—●) and -untreated (○ – – ○) castrated mice. For each transplant generation the top level in the $T_3$ curve (*i.e.* the maximum relative amount of cell clumps: see Fig. 2) was taken as the degree of maximal agglutination.

concentrations of the lectin caused marked clumping of cells. The concentration of Con A, required for a 50% decrease in the amount of single cells was similar (2–3 µg/ml) for all the transplant generations studied, except for P13 and P14 (5–6 µg/ml). If the number of single cells ($T_1$–$T_2$) in the absence of Con A was taken as 100, the maximum number of Con A-induced clumps of 4 or more cells obtained from tumours of the 1st, 10th and 13th transplant generations was 2·4 (Fig. 2A), 4·3 (Fig. 2B) and 9·8 (Fig. 2C), respectively. After these maxima were reached, the number of clumped cells usually remained fairly constant when still more Con A was added, but a slight drop in clumping was sometimes found at very high concentrations (100 µg/ml) of the lectin.

In Fig. 3 the number of clumped cells is plotted for each transplant generation studied. Con A-mediated agglutination was low in hormone-dependent transplant generations 1–9, but increased in hormone-responsive transplant generations 10–13, with a further increase in hormone-independent transplant generations 13–16. A sharp peak in cytoagglutination occurred with transplant generation 13. This peak was observed with both hormone-treated and -untreated tumours.

### DISCUSSION

The data presented here show that changes occur in the lectin-mediated agglutinability of GR mouse mammarytumour cells when these are serially transplanted. The agglutinability was low for hormone-dependent tumour cells, but increased with diminishing response of the tumours to hormones. Since the decrease of hormone responsiveness of GR mammary tumours is paralleled by a decrease in prolactin receptors (Costlow *et al.*, 1977) and, since Con A inhibits prolactin binding in some systems (Costlow & Gallagher, 1977), it seems possible that the loss of prolactin-binding and other recognition sites on the cell membrane is due to an increased expression and masking effect of Con A receptors, which in turn might be related to the increase in lectin-mediated agglutinability. However, a direct relationship between agglutinability and hormone dependency is difficult to establish, since our data also indicate a change in the histology of the tumour, which itself might influence the Con A-mediated agglutinability of the cells. It seems possible that the very high agglutinability of transplant generation 13 was related to this tumour being an anaplastic carcinoma, consisting of loosely arranged cells. It is possible that, because these cells were not as interconnected as cells of other transplant generations, they show an enhanced expression and/or avidity of receptors for Con A.

In conclusion, our results suggest that, though hormone-independent GR mouse mammary-tumour cells in general have a higher Con A-mediated agglutinability than their hormone-dependent counterparts, it is questionable whether this reaction can be used as a marker for hormone dependency, because changes in tumour histology may also cause changes in agglutinability.

The authors are indebted to Mr H. A. M. Hilkman, Mr C. C. J. De Goeij and Mrs S. G. Evers-Klaver for their technical assistance.

REFERENCES

BRIAND, P. & DAEHNFELDT, J. L. (1973) Enzyme patterns of glucose catabolism in hormone-dependent and -independent mammary tumours of GR mice. *Eur. J. Cancer*, **9**, 763.

BURGER, M. M. (1969) A difference in the architecture of the surface membrane of normal and virally transformed cells. *Proc. Natl Acad. Sci., U.S.A.*, **62**, 994.

BURGER, M. M. (1970) Proteolytic enzymes initiating cell division and escape from contact inhibition of growth. *Nature*, **227**, 170.

COSTLOW, M. E. & GALLAGHER, P. E. (1977) Concanavalin A induced alterations in $^{125}$I-labelled prolactin binding. *Biochem. Biophys. Res. Commun.*, **77**, 905.

COSTLOW, M. E., SLUYSER, M. & GALLAGHER, P. E. (1977) Prolactin receptors in mammary tumors of GR mice. *Endocr. Res. Commun.*, **4**, 285.

INBAR, M., BEN-BASSAT, H. & SACHS, L. (1971) A specific metabolic activity on the surface membrane in malignant cell-transformation. *Proc. Natl Acad. Sci., U.S.A.*, **68**, 2748.

INBAR, M., BEN-BASSAT, H. & SACHS, L. (1972) Membrane changes associated with malignancy. *Nature (New Biol.)*, **236**, 3.

INBAR, M. & SACHS, L. (1969) Interaction of the carbohydrate binding protein concanavalin A with normal and transformed cells. *Proc. Natl Acad. Sci., U.S.A.*, **63**, 1418.

QUIST, R., PALIN, C. & HEIBERG, I. (1976*a*) Transfer RNA and aminoacyl tRNA synthetases in hormone-dependent and -independent mammary tumors of GR mice. I. Comparative study of the amino acid accepting capacity of the tRNA's in the presence of the homologous and heterologous enzymes. *Cancer Biochem. Biophys.*, **1**, 215.

QUIST, R., PALIN, C. & HEIBERG, I. (1976*b*) Transfer RNA and aminoacyl tRNA synthetases in hormone-dependent and -independent mammary tumors of GR mice. II. Isoacceptor tRNA's in hormone-dependent and -independent mammary tumors of GR mice. *Cancer Biochem. Biophys.*, **1**, 317.

SLUYSER, M., EVERS, S. G. & DE GOEY, C. C. J. (1976) Sex hormone receptors in mammary tumors of GR mice. *Nature*, **263**, 386.

SLUYSER, M., NOUWEN, T., HILGERS, J. & CALAFAT, J. (1977) Levels of mammary tumor virus in hormone-dependent and -independent mouse mammary tumor cells. *Cancer Res.*, **37**, 1986.

SLUYSER, M. & VAN NIE, R. (1974) Estrogen receptor content and hormone-responsive growth of mouse mammary tumors. *Cancer Res.*, **34**, 3253.

SMETS, L., VAN BEEK, W. P. & VAN NIE, R. (1977) Membrane glycoprotein changes in primary tumors associated with autonomous growth. *Cancer Lett.*, **3**, 133.

THORPE, S. M. (1976) Increased uptake of iodide by hormone-responsive compared to hormone-independent mammary tumors in GR mice. *Int. J. Cancer*, **18**, 345.

VAN BLITTERSWIJK, W. J., WALBORG, E. F., FELTKAMP, C. A., HILKMANN, H. A. M. & EMMELOT, P. (1976) Effect of glutaraldehyde fixation on lectin-mediated agglutination of mouse leukaemia cells. *J. Cell Sci.*, **21**, 579.

WIEPJES, G. J. & PROP, F. J. A. (1970) Improved method for preparation of single-cell suspensions from mammary glands of adult virgin mouse. *Exp. Cell Res.*, **61**, 451.